# Factors affecting affect cardiovascular health in Indonesian HIV patients beginning ART

**DOI:** 10.1186/s12981-017-0180-9

**Published:** 2017-08-31

**Authors:** Birry Karim, Ika Praseya Wijaya, Rizky Rahmaniyah, Ibnu Ariyanto, Shelley Waters, Riwanti Estiasari, Patricia Price

**Affiliations:** 1 Cardiology Division, Internal Medicine, Universitas Indonesia, Jakarta, Indonesia; 2 Cipto Mangunkusomo Hospital, Jakarta, Indonesia; 3 Virology and Cancer Pathobiology Research Center, Universitas Indonesia, Jakarta, Indonesia; 4 School of Biomedical Sciences, Curtin University, Bentley, WA Australia; 5 Department of Neurology, Universitas Indonesia, Jakarta, Indonesia

**Keywords:** Anti-retroviral therapy, Cardiovascular disease, Cytomegalovirus, HIV

## Abstract

**Background:**

We present a small longitudinal study of how demographic factors and persistent burdens of HIV and cytomegalovirus (CMV) influence cardiovascular health in young adults beginning ART in an inner-city clinic in Jakarta, Indonesia.

**Methods:**

ART-naïve HIV patients [n = 67; aged 31 (19 to 48) years] were enrolled in the JakCCANDO Project. Echocardiography and carotid Doppler ultrasonography were performed before ART (V0) and after 3, 6, and 12 months (V3–12). Antibodies reactive with CMV lysate or IE-1 protein were assessed at each timepoint and CMV DNA was identified at V0.

**Results:**

Markers of adverse cardiovascular prognosis [left ventricular mass index, ejection fraction and carotid intimal media thickness (cIMT)] were similar to healthy controls, but increased at V12. Internal diameters of the carotid arteries and systolic blood pressure correlated with HIV disease severity at V0, but cardiac parameters and cIMT did not. E/A ratios (left ventricular diastolic function) were lower in patients with CMV DNA at V0, but this effect waned by V6. Levels of antibody reactive with CMV IE-1 correlated inversely with CD4 T cell counts at V0, and levels at V6–V12 correlated directly with the right cIMT.

**Conclusions:**

Overall the severity of HIV disease and the response to ART have only subtle effects on cardiovascular health in this young Asian population. CMV replication before ART may have a transient effect on cardiac health, whilst antibody reactive with CMV IE-1 may mark a high persistent CMV burden with cumulative effects on the carotid artery.

## Introduction

Several studies have demonstrated accelerated age-related syndromes, such as vasculopathy, in HIV patients assessed in “western” settings. Most have addressed patients over 40 years of age, with consideration to traditional risk factors such as smoking, diet and exercise. In this context, the consensus view ascribes vascular pathology to systemic inflammation in untreated patients, where this declines on antiretroviral therapy (ART) and metabolic factors become dominant [1–3]. Cardiac parameters are less well studied, but Caucasian and African American patients receiving ART had a higher prevalence of diastolic dysfunction and higher left ventricular mass indices (LVMI) than healthy controls. These differences were not readily explained by differences in traditional risk factors and were independently associated with HIV infection [4, 5]. However, ART changes patterns of cardiac dysfunction from myocarditis [caused by HIV itself or opportunistic infections including cytomegalovirus (CMV)] to syndromes mediated by autoimmunity and antiretroviral drug toxicities [6]. Hence cardiovascular risk in HIV patients on ART is more effectively predicted by the D:A:D algorithm based on Framingham scores and critical anti-retroviral drugs, than by Framingham scores alone [7]. Simulated interventions applied to an Asian population found smoking cessation had the greatest potential impact on 5-year predicted risks of cardiovascular disease, approximating the effect of switching from abacavir to an alternate antiretroviral drug [8]. However abacavir is now used sparingly and the standard regimes cause minimal cardiovascular toxicity [9].

Several studies link a high burden of CMV with accelerated T-cell differentiation and cardiovascular disease in HIV patients stable on ART (e.g. [10]). One study showed that CMV prophylaxis can reduce immune activation [11], and may thus reduce vascular inflammation. Our study of Caucasian Australian patients stable after more than 2 years on ART correlated levels of antibody reactive with a lysate of CMV-infected fibroblasts with D:A:D scores [12]. However, studies of the younger HIV patients who predominate in Asian cohorts are rare, and the roles of persistent opportunistic infections (including CMV and tuberculosis; [6]) remain unclear. Here we address the effect of CMV and ART directly through standard measures of cardiovascular function applied to young adult patients beginning treatment with moderately advanced HIV disease in an inner city clinic in Jakarta, Indonesia. Patients from this clinic have high titres of antibody reactive with CMV [13] and many have tuberculosis.

## Materials and methods

### Study population

The JakCCANDO Project is a comprehensive survey of clinical and immunological responses to ART undertaken in the outpatient clinic of Cipto Mangunkusumo Hospital (Jakarta, Indonesia). We enrolled 82 ART-naïve HIV patients in 2013–2014 with <200 CD4 T-cells/µl. The study was approved by Universitas Indonesia, Cipto Mangunkusumo Hospital and Curtin University ethics committees. Written consent was obtained from each subject. Examinations were performed before ART initiation (V0) and at months 3, 6 and 12 (V3, V6, V12). Subjects were also tested for pulmonary tuberculosis (chest X-ray and sputum acid bacilli smear) at V0. Plasma HIV RNA loads were determined using AmpliPrep/COBAS^®^ TaqMan^®^ HIV-1 Tests (version 2.0) and CD4 T-cell counts were determined using standard flow cytometric techniques.

### Cardiovascular assessments

Echocardiography and vascular Doppler examinations used an ESAOTE ultrasonography unit (Genova, Italy), with a LA522E ultrasound probe to evaluate the carotid artery and a PA230E probe to evaluate the heart. For cardiac examinations, the probe was positioned on the chest wall to gain B-mode and M-mode views. Parameters recorded included the Ejection Fraction (EF; the percentage of blood leaving the heart at each contraction) and the E/A ratio [ratio of the early (E) to late (A) ventricular filling velocities marking the ability of the left ventricle to fill between contractions]. LVMI were calculated using the Devereux formula [14] incorporating diastolic measurements of the left ventricular internal diameter (LVID), interventricular septal thickness (IVST) and posterior wall thickness (PWT): LVMI (g/m^2^) = (1.04 [(IVST + LVID + PWT)^3^ − LVID^3^] − 14)/height squared. Carotid Doppler sonography was used to evaluate arterial circulation using B-mode, color flow and velocity measurements. The outcome is expressed as carotid intimal medial thickness (cIMT) and as the internal diameter of the common carotid artery measured at the same site when the artery was in the diastolic phase. Blood pressure (BP) was recorded with the subject lying down. All assessments were made by a single qualified operator (B.K.).

### Quantitation of the burden of CMV

CMV-reactive CMV IgG was quantified using 96-well plates coated with a lysate of human foreskin fibroblasts (HFF) infected with CMV strain AD169, or with CMV IE-1 (immediate early 1) prepared in *E. coli* (Miltenyi Biotech; Cologne, Germany). CMV-reactive antibodies were quantitated relative to a standard assigned a value of 1000 arbitrary units (AU). To detect CMV DNA, primer and probe sequences targeting the UL54 gene (kindly shared by Andrew Davison, University of Glasgow) were optimized using DNA extracted from HFF infected with CMV AD169, diluted in a tenfold series to generate a standard curve. Total DNA was extracted from blood neutrophils with QIAmp DNA Blood Mini kits (Qiagen, Hilden, Germany). Reactions were performed in a total volume of 20 µl containing 10 µl Universal PCR Master mix with ROX reference dye and uracil N′-glycosylase (Applied Biosystems, Foster City, CA), 0.8 µl 10 µM primers, 0.6 µl 5 µM *Taq*Man probe and 5 µl DNA. Cycling conditions were 2 min at 50 °C, 10 min at 95 °C, 40 cycles of 15 s at 95 °C and 60 s at 60 °C. Results were normalized against the single-copy housekeeping gene β2-microglobulin [15].

### Statistical analyses

Clinical and laboratory variables were assessed using non-parametric statistics as many failed the D’Agostino and Pearson omnibus normality test at some or all time points. This included Wilcoxon and Mann–Whitney tests for group wise comparisons (paired and unpaired data, resp.), and Spearman’s correlations (GraphPad Prism version 6.0 for Mac OS, La Jolla, CA). Comparisons achieving p < 0.05 are interpreted as significant differences, but p < 0.10 is noted when part of a consistent pattern. Bonferroni corrections were not made as the study is primarily descriptive and will require replication in an independent cohort.

## Results

Study patients were drawn from the 82 individuals enrolled in the JakCCANDO cohort. By V12, six had died, four had withdrawn from ART, two were pregnant and six were lost to follow up. Data are presented for the 46 male and 21 female patients [median (range) age 31 (19 to 48) years], who provided at least one follow-up assessment of vascular health (carotid diameter and cIMT) and cardiac function (E/A ratio, EF, LVMI) (see Table 1). All JakCCANDO patients received triple therapy including lamivudine, zidovudine, nevirapine, stavudine, efavirenz and/or tenofovir. None were prescribed abacavir, so the drugs administered have no confirmed association with vascular pathology [8]. Healthy controls (7 males, 4 females) aged 30 (22 to 38) years were assessed once, and data were compared with published endpoints used in clinical care.

**Table 1:** Cardiovascular assessments during the first year on ART

n	Pre-ART	3-months	6-months	12-months	Healthy controls
	67	60	54	55	11
CD4 T-cells/μl	63 (2–199)	181 (7–601)^b^	202 (6–516)^b^	285 (44–763)^b^	–
HIV RNA^a^	5.14 (2.91–6.68)	1.89 (0–5.23)^b^	0 (0–5.33)^b^	0 (0–6.32)^b^	–
BMI (kg/m^2^)	19.6 (13.2–36.9)	20.5 (14.7–39.8)	21.7 (16.0–40.1)^b^	23.0 (13.4–40.2)^b^	18.5–25^d^
BP (Systolic)	110 (100–150)	110 (100–130)	110 (110–160)	120(100–146)^b^	90–120^d^
BP (Diastolic)	80(70–100)	80 (60–90)	80 (60–100)	80(60–90)	60–90^d^
EF (%)	68 (51–84)^c^	69 (50–83)	70 (61–80)	70 (50–79)^b^	71 (53–77)
E/A Ratio	1.3 (0.6–4.7)^c^	1.3 (0.8–1.9)	1.3 (0.8–1.9)	1.3 (1.1–1.8)	1.4 (1.0–1.9)
LVMI	94 (30–177)	99 (52–187)	100 (57–217)^c^	102 (47–222)^c^	83 (48–125)
cIMT (right, mm)	0.58 (0.39–0.64)	0.58(0.38–0.77)	0.57(0.45–0.90)	0.70(0.46–1.0)^b^	0.58 (0.39–0.83)
cIMT (left, mm)	0.57 (0.32–0.89)	0.57(0.39–0.77)	0.51(0.32–0.89)	0.65(0.45–0.96)^b^	0.58 (0.45–0.70)
Diameter (right)^e^	6.4 (4.0–7.9)	6.2 (4.3–9)	5.9 (4.7–8.3)	6.2 (5.1–8.8)	(5–7.5)^d^
Diameter (left)^e^	6.1 (3.5–8.1)	6.2 (4.1–7.5)	6.0 (4.5–8.0)	5.8 (4.1–8.0)	(5–7.5)^d^

All data are presented as median (range)

BMI, body mass index; BP, blood pressure in mmHg; EF, ejection fraction; E/A ratio, early/late ventricular filling; cIMT, carotid artery intimal thickness; LVMI, Left Ventricular Mass Index. See "Materials and Methods" for details

^a^Log_10_ copies/ml

^b^Significantly different from V0, Wilcoxon test, p < 0.05

^c^Significantly different from healthy controls, Mann Whitney test, p < 0.05

^d^Normal range used for clinical care in Jakarta

^e^Diameter of the carotid artery (mm)

cIMT values and vessel diameters displayed direct correlations between the right and left arteries (r = 0.39 to 0.59, p = 0.002 to <0.0001), but the right and left were not identical at any timepoint and were significantly different at V12 (cIMT, p = 0.01; diameter, p = 0.0005). Hence both are presented. Factors affecting cardiovascular outcomes are discussed below.

### Gender and age

Male and female patients were similar in age, BMI and HIV RNA levels (data not shown), but female patients had slightly slower recovery on ART evidenced by lower CD4 T-cell counts at V6 [134 (36 to 339) vs 208 (6 to 516) cells/µl, p = 0.016]. Male and female patients did not differ in cIMT at any time, but males had slightly higher diameters of their carotid arteries. This was clearest at V0 and V12 (p = 0.08 to 0.008). Males had slightly lower EF values than females. This was marginal at V6 (p = 0.11) and significant at V12 [0.67 (0.50 to 0.79) vs 0.72 (0.65 to 0.78), p = 0.05]. LVMI values were slightly higher in males, with a significant difference at V0 [98 (30 to 177) vs 83 (51 to 129), p = 0.03] diminishing by V3 (p = 0.10), and not evident thereafter. As these differences were small and in accord with general practise, data from males and females were pooled unless otherwise noted.

Increasing age was associated with higher cIMT values. The association was weak at V0 [right: r = 0.26, p = 0.04, left: r = 0.12, p = 0.36], perhaps reflecting the overlaid influence of HIV. However it was consistent thereafter (e.g.; at V6, right: r = 0.36, p = 0.01, left: r = 0.33, p = 0.03). Accordingly, E/A ratios (assessing left ventricular function) declined with age, with significant negative correlations at V0, V3 and V6 (r = −0.25 to −0.3, p = 0.03 to 0.04). CD4 T-cell counts and blood pressure did not associate with patient age.

### Smoking and alcohol consumption

37% of patients (24 males, 1 female) were current smokers. As this represents a clear gender imbalance (Fisher’s exact test, p < 0.0001), effects of smoking were analyzed in males. No measures of HIV disease or cardiovascular function were influenced by smoking (p > 0.19 throughout).

No females and 22 males admitted some consumption of alcohol, most saying this was in the past. Cardiovascular parameters were similar in patients who had ever or never consumed alcohol (p > 0.16 for all comparisons). However, consumption is expected to be low and may be under reported in a Muslim country.

### Tuberculosis and systemic inflammation

51% of patients had a diagnosis of pulmonary tuberculosis before ART. They had slightly higher baseline log_10_ HIV RNA levels [5.3 (3.7 to 6.7) vs 4.8 (2.9 to 6.4), p = 0.05] and lower BMI [19.1 (13.2 to 25.0) vs 19.9 (15.6 to 36.9), p = 0.02]. Cardiovascular parameters were unaffected, with the exception of the diameter of the carotid artery which was lower at V0 in patients with tuberculosis (right: p = 0.06, left: p = 0.0007), with smaller changes thereafter. Plasma CRP levels were elevated relative to healthy controls and unchanged on ART (Table 1). Levels were not associated with any parameters of cardiovascular disease (p > 0.10) and only marginally increased by tuberculosis (e.g. p = 0.06 at V6).

### BMI

A low BMI is the hallmark of HIV disease, so it is not surprising that BMI values increased on ART (V0 vs V12; p < 0.0001). Accordingly, the BMI at baseline was directly related to CD4 T-cell counts at all time points (r = 0.21 to 0.33, p = 0.09 to 0.01). The weak inverse association between baseline HIV RNA levels and BMI at V0 (r = −0.21, p = 0.08) strengthened when the BMI was re-assessed at V3 and V6 (r = −0.31, p = 0.02 at each time).

Associations between BMI and cardiovascular parameters were variable but generally strengthened on ART. BMI correlated weakly with blood pressure throughout, but the link was clearest at V12 (r = 0.47, p = 0.0003) when the systolic pressure had increased relative to V0 (p = 0.02). Positive associations between BMI and the diameters of the both carotid arteries were significant at most timepoints (r = 0.26 to 0.53, p = 0.01 to < 0.0001).

Many studies utilise waist hip ratios as an alternative to BMI. These have been validated as marking cardiovascular health in older HIV patients stable on ART, but are less clearly affected by HIV than BMI in younger patients [16]. Overall the importance of waist hip ratios in prediction of CVD in young (mostly slim) Asian patients beginning ART is unproven.

### The severity of HIV disease and the response to ART

The diameter of the carotid artery correlated directly with CD4 T-cell counts at V0 and V3 (r = 0.21 to 0.37, p = 0.11 to 0.004) and inversely with baseline log_10_ HIV RNA levels (r = −0.27, p = 0.04). This is in accord with its association with tuberculosis, mentioned previously. cIMT values increased after 12 months on ART (Wilcoxon paired test, p < 0.0001; see Table 1), but did not correlate with CD4 T-cell counts or HIV RNA loads (data not shown).

Systolic blood pressures recorded at V0 correlated with CD4 T-cell count at that time (r = 0.33, p = 0.006), with weaker but positive correlations thereafter. Moreover systolic pressures on ART correlated inversely with the HIV RNA load at V0 (r = −0.24 to −0.35, p = 0.06 to 0.01), with no consistent associations with the HIV RNA levels at later time points. This suggests a long-term effect of the viral set-point irrespective of the response to ART.

The EF value at V0 was lower than healthy controls (p = 0.03, Table 1). Although EF values increased (p = 0.04) by V12, they were not correlated with CD4 T-cell counts or HIV RNA levels at any time. The E/A ratio correlated with the HIV RNA at V0 (r = 0.35, p = 0.004), but did not change on ART. LVMI values were generally high in the patients and increased further to become higher than the control values at V6 and V12 (p = 0.02 and p = 0.05, resp.). Elevated LVMI values may reflect immune recovery, as values recorded at V3 correlated with CD4 T-cell counts at all time points (r = 0.27 to 0.42, p = 0.03 to p = 0.002).

### The burden of CMV

CMV DNA was detectable at V0 in 30/64 (47%) patients who provided cardiovascular data. The presence of CMV DNA did not associate with HIV RNA levels (Mann–Whitney, p = 0.53), CD4 T-cell counts (p = 0.31) or CRP levels (p = 0.81), but was more common in patients with pulmonary tuberculosis (χ^2^, p = 0.04). E/A ratios were slightly lower in patients with CMV DNA at baseline [1.20 (0.88 to 1.78) vs 1.36 (0.77 to 2.14); p = 0.03], with a similar pattern at V3. However E/A ratios increased in CMV DNA positive patients by V6, so the two groups were similar at that time [1.37 (0.84 to 1.90) vs 1.32 (1.05 to 1.71), p = 1.0]. Hence CMV DNA in buffy coats may mark transient ill health or CMV myocarditis. The finding aligns with the correlation between the E/A ratio and HIV RNA at V0 (r = 0.35, p = 0.004) mentioned earlier. No other markers of cardiovascular health (including BMI) aligned with CMV DNA at V0.

Levels of CMV reactive antibody rose on ART, stabilising by V6 (Table 1) at levels higher than in healthy controls (CMV lysate, p = 0.05; CMV IE-1, p = 0.003). However values recorded in patients at different timepoints were tightly correlated (p < 0.001), so high or low responses were a stable feature of an individual.

The presence of CMV DNA at baseline increased levels of antibody reactive with CMV IE-1 at V0 (p = 0.04), but had no other effect on CMV antibody levels. Levels of CMV IE-1 antibody at V3 and V6 correlated with levels of CRP at V0 and V3 (r = 0.21 to 0.26, p = 0.10 to 0.03) and with low CD4 T-cell counts at V0 (r = 0.34 to 0.40; p = 0.01 to 0.002) (Table 2). This links CMV IE-1 antibody with advanced HIV disease pre-ART. In contrast levels of CMV lysate antibody increased with age (r = 0.23 to 0.28, p = 0.07 to 0.02). Levels of antibody reactive with CMV IE-1 or CMV lysate at V6 and V12 correlated inversely with BMI at V0 (r = −0.27 to −0.36; p = 0.01 to 0.03). These associations suggest a high persistent CMV burden in patients who began ART with advanced HIV disease and systemic inflammation, where bursts of CMV replication may be detected more effectively by levels of IE-1 antibody than CMV DNA.

**Table 2:** Levels of CMV-reactive antibody assessed on ART correlate with changes to the right carotid artery

R	Visit	CMV lysate antibody				CMV IE-1 antibody			
		V0	V3	V6	V12	V0	V3	V6	V12
Age		**0.28**	**0.23**	**0.25**	**0.27**	0.10	0.11	0.15	0.14
CD4 T-cells	V0	0.18	−0.17	−*0.23*	−0.19	−0.19	**−** ***0.34***	**−** ***0.35***	**−** ***0.40***
	V3	0.04	−0.09	−0.10	−0.08	−0.15	−0.15	0.01	0.02
	V6	0.10	−0.01	−0.05	−0.02	−0.13	−0.13	−*0.24*	**−** ***0.26***
	V12	0.09	−0.07	−0.05	0.01	−0.10	−0.13	−0.04	−0.10
HIV RNA	V0	−0.22	−0.09	−0.01	0.02	0.00	0.07	−0.17	−0.19
	V3	−0.16	0.05	0.05	0.03	−0.11	0.02	−0.17	−0.17
	V6	−0.15	0.03	0.01	0.02	−0.15	−0.07	−0.14	−0.13
	V12	−0.16	−0.05	−0.07	−0.20	−0.04	−0.19	**−** ***0.33***	−*0.25*
Diameter right	V0	0.03	−0.03	−0.06	−0.21	−0.14	−0.18	−0.11	−*0.25*
	V3	0.11	0.11	−0.10	−*0.27*	−***0.28***	−*0.27*	**−** ***0.30***	**−** ***0.46***
	V6	0.00	−0.15	−*0.27*	**−** ***0.32***	−0.09	−0.12	−0.13	−0.16
	V12	0.02	−0.18	**−** ***0.31***	**−** ***0.29***	0.04	−0.12	−0.22	−0.19
cIMT right	V0	0.08	0.17	0.18	0.21	−0.03	0.10	**0.27**	**0.31**
	V3	**0.27**	0.15	0.14	0.12	0.11	0.10	0.10	0.16
	V6	0.18	0.21	0.23	0.21	0.07	0.14	**0.29**	**0.30**
	V12	0.00	−0.06	−0.02	0.07	0.06	0.03	0.21	**0.26**
Diameter left	V0	0.07	0.01	−0.06	−0.14	−0.10	−0.21	−0.21	−*0.27*
	V3	−0.08	0.01	−0.01	−0.08	−0.10	−0.14	0.01	−0.06
	V6	−0.03	−0.16	−0.24	−0.17	0.02	0.02	−0.12	−0.01
	V12	0.08	−0.07	−0.06	−0.06	0.05	0.03	−0.05	−0.10
cIMT left	V0	−0.12	0.02	0.16	0.10	−0.11	0.01	**0.23**	0.16
	V3	0.14	0.03	0.03	0.02	0.02	−0.07	−0.12	−0.02
	V6	−0.09	0.07	0.10	0.15	0.10	0.08	0.11	0.19
	V12	0.10	0.12	0.05	0.04	0.10	−0.02	0.10	0.03

Spearman’s correlation coefficients comparing levels of antibody reactive with CMV lysate or CMV IE-1 protein with measures of HIV disease and the health of the carotid artery. As a visual aid, positive correlations are marked in bold underlined (p < 0.05) and bold font (p < 0.10), and negative correlations are marked in bold italics (p < 0.05) and italics (p < 0.10)

Levels of antibody reactive with CMV lysate recorded at V6 and V12 correlated inversely with the diameter of the right carotid artery at V6 and V12. Levels of CMV IE-1 antibody recorded at any time also correlated inversely with the diameter of the right artery (notably with readings from V3) and displayed direct correlations with right cIMT values (Table 2). No correlations between levels of either CMV antibody and the left carotid artery, E/A ratios, EF or LVMI values achieved p < 0.05 (Table 2, data not shown).

## Discussion

Our study of cardiovascular parameters in a young adult population entering treatment in Jakarta shows subtle changes over the first year on ART. Whilst the small size of our cohort precludes multivariable analyses and corrections for multiple comparisons, it is novel and interesting as a preliminary descriptive study. Low CD4 T-cell counts, high HIV RNA levels and/or the presence of pulmonary tuberculosis at V0 associated with low BMI, smaller diameter of the carotid artery and/or higher systolic blood pressure, but not consistently with markers of cardiovascular health assessed at any time. EF, LVMI and cIMT rose slightly after 12 months on ART, but other parameters were stable. Accordingly at V3, LVMI values correlated directly with CD4 T-cell counts. The rise in EF suggests improved cardiac function, but the higher cIMT and LVMI values suggest a deterioration consistent with poor cardiac outcomes. The latter contrast with a study of Nigerian HIV patients where cardiac disease associated with lower CD4 T-cell counts [17]. However it was a cross-sectional study merging treated and untreated patients, so changes on ART were not captured.

We assessed the burden of CMV in three ways. Levels of CMV DNA reflect active viral replication and may change over days or weeks. We did not screen all samples from later timepoints, but 12/17 samples positive at V0 and re-tested at V3 were positive (Ariyanto et al., unpublished data). Here high CMV IE-1 antibody levels on ART were a feature of patients with low CD4 T-cell counts, a low BMI and elevated CRP at V0/V3. The association between CMV lysate antibodies and CD4 T-cells at V0 was weak, and levels increased with age. These patterns were also seen in our assessments of Australian patients stable on ART [12, 18]. Hence levels of antibody reactive with CMV IE-1 protein on ART reflect frequent reactivations consequent to advanced HIV disease with immune activation pre-ART. As levels of both antibodies rise on ART, levels recorded before ART may underestimate the burden of CMV at that time. By corollary, levels of CMV-reactive antibody assessed on ART may be a better metric for the burden of CMV.

CMV DNA at baseline affected E/A ratios, indicating abnormal ventricular filling between contractions, and hence may mark transient ill-health or myocarditis caused by CMV or other co-infections. This resolved on ART. Patients with CMV DNA were also more likely to have pulmonary tuberculosis. Whilst extra-pulmonary tuberculosis was not recorded, disseminated infections are not uncommon in the clinic population and mycobacterial pericarditis may affect ventricular function [6]. Abnormal ventricular filling measured by low E/A ratios carries an increased risk of diastolic heart failure.

In addition to these acute effects, levels of antibody reactive with CMV lysate recorded at V6 and V12 were inversely proportional to the diameter of the right carotid artery. Similarly we found an inverse relationship between levels of the CMV IE-1 antibody at any time and the diameter of the right artery at V3. Importantly, levels of CMV IE-1 antibodies also correlated directly with cIMT values as both parameters rose on ART. The left carotid artery showed no equivalent correlations.

Selective associations with the right carotid artery may illuminate the pathogenic mechanisms invoked by CMV. Left cIMT values are typically higher than the right in healthy adults over 40 years of age, with little difference in younger individuals [19]. High cIMT in the left artery may reflect intimal hyperplasia or medial hypertrophy, arising from increased haemodynamic stress at that side [20]. Atherosclerotic progression may be faster on the left side, and more tightly linked to the deposition of triglycerides [21]. Hemorrhage, lipid deposition and fibrosis were most prevalent in plaques of the left artery, whilst plaques in the right artery were more frequently calcified and stable in a cohort aged 72 ± 10 years [22]. Most individuals in JakCCANDO were below 40 years of age, so it is not surprising that their left cIMT values were not elevated. We found no studies of CMV in HIV patients where the right and left arteries were presented separately, but two studies where the values were averaged could not link CMV antibody levels with cIMT [23, 24]. The authors had sought a link in view of studies associating other measures of CMV load with cardiovascular risk (e.g. [10–12])—indeed we find a correlation here between CMV IE-1 antibodies and the right artery. Further studies should asses the right and left arteries are separately after defined and longer periods on ART. Assays of endothelial function (vascular elasticity) are also needed as they may better mark the effects of CMV [23, 25].

Overall the severity of HIV disease and the response to ART have only subtle effects on cardiovascular health in this young Asian population but these do not resolve on ART. As Cipto Mangunkusomo is a tertiary referral hospital, it is current practice for HIV patients to be referred directly to a cardiologist only if they have acute coronary syndrome or a stroke, and indirectly for echocardiography only if they have symptoms or an enlarged heart. Our data suggest continued monitoring would confer clinical benefits.

## Conclusions

Measures of CMV antibodies and DNA display distinct associations with cardiovascular parameters. We propose antibodies reactive with CMV IE-1 as a marker of vascular pathology as levels are relatively stable and correlate with changes to the right carotid artery. Although CMV IE-1 antibodies associate with starting ART with a low CD4 T-cell count, the count itself did not predict changes to either artery.

## Abbreviations

ART: antiretroviral therapy
CMV: cytomegalovirus
cIMT: carotid intimal media thickness
EF: ejection fraction
E/A ratio: ratio of the early (E) to late (A) ventricular filling velocities
IE: immediate early gene of CMV
LVMI: left ventricular mass index
V0: visit at baseline (0 months on ART)
